# Calcium, Vitamin D, and Aging in Humans

**DOI:** 10.3390/nu16233974

**Published:** 2024-11-21

**Authors:** Ligia J. Dominguez, Stefano Gonnelli

**Affiliations:** 1 Department of Medicine and Surgery, “Kore” University of Enna, 94100 Enna, Italy; 2 Section of Internal Medicine, Department of Medicine, Surgery and Neuroscience, University of Siena, 53100 Siena, Italy; stefano.gonnelli@unisi.it

There is currently a growing awareness that nutritional factors have major impacts on the risk of age-associated chronic non-communicable diseases and mortality [1]. Two of these nutrients, calcium and vitamin D, have been particularly implicated in maintaining musculoskeletal health, which are increasingly common conditions at different stages of life but particularly in old age. In fact, adults and older persons with inadequate dietary calcium intake or insufficient vitamin D levels have an increased risk of osteoporosis and fragility fractures, which are continuously increasing worldwide [2]. There is evidence that the supplementation of these nutrients in frail older adults living in long-term care facilities reduces the risk of hip and other fragility fractures [3,4,5]. In addition, vitamin D supplementations may improve muscle function and contribute to decreasing the risks of falling in older adults with deficiency of this nutrient [6,7,8]. Moreover, vitamin D may have biological effects well beyond the skeleton, and several studies support an association between low vitamin D concentrations and an increased risk of dementia, diabetes, cancer, and infectious diseases [9,10,11]. However, further well-designed studies are needed to better define the role of vitamin D in the prevention and treatment of these diseases in older adults. This Special Issue brings together some articles with recent and relevant data on these two nutrients that are essential for human health at all ages, and, in particular during aging.

One of the articles explored the efficacy of the recommendations promoted for a decade in Poland aiming to prevent vitamin D deficiency in older adults [12]. Lech et al. analyzed data from community-dwelling participants of the PolSenior 2 study who were admitted to a geriatrics ward to evaluate the prevalence of vitamin D deficiency and the adherence to recommended health-promoting behaviors (i.e., taking vitamin D supplements). The authors found that among 240 participants, over two-thirds (67.5%) had inadequacy of 25OHD levels (<30 ng/mL), and 15% had profound deficiency (<10 ng/mL) of this fundamental nutrient. Only 18.3% of participants were on vitamin D supplements before admission, and, as expected, insufficiency was significantly more prevalent in those without using supplements (70.9% vs. 52.3%) and in obese participants (body mass index [BMI] ≥ 30 kg/m^2^). Furthermore, obese participants had over twice the risk of vitamin D deficiency compared to those not receiving supplements. Thus, despite recommendations, the prevalence of vitamin D deficiency is remarkably high in community-dwelling Polish older adults, and targeted interventions and education for older adults, caregivers, and health providers are still needed to fill this gap. Currently, it could be hypothesized that vitamin D deficiency in children no longer exists in Western countries. As a comparison with what occurs in adulthood and in old age, the study by Van de Walle and coworkers evaluated the vitamin D status among a large sample (n = 14,887) of Belgian children aged 0–18 years between 2014 and 2021 [13]. Even in this young group, 17.7% were vitamin D severely deficient (<12 ng/mL), 25.2% were insufficient (12–20 ng/mL), and another large proportion (28.3%) was borderline (20–30 ng/mL). Sufficient levels (>30 ng/mL) were met in only 28.8% of the sample. Adolescents aged 13–18 years had the highest prevalence of severe vitamin D deficiency (24.9%). Thus, vitamin D deficiency and insufficiency are also remarkably prevalent in this young age group. The authors warn that Belgian health authorities should be aware of these results, as the current Belgian recommendation suggests ceasing vitamin D supplementation at the age of six. These children, who will become adults and old people in the future, could be exposed to a lifelong vitamin D deficiency with worrying consequences for their health in later life.

Cianferotti and coworkers reviewed available randomized controlled trials, prospective and intervention studies, and systematic reviews on the screening, assessment, and management of nutritional and vitamin D status and calcium intake in patients before and after having a hip fracture [14]. The in-depth review of the studies available in recent years, with all their contradictions, collectively suggests that targeted nutritional interventions, including calcium and protein intake through diet or supplements, together with rehabilitation programs, can significantly contribute to improved recovery and mobility after hip fractures. Such strategies can also contribute to the prevention of future new fractures by improving overall bone and skeletal muscle health. The review also emphasizes the role of fracture liaison services, which represent the most reliable model of management for hip fracture patients.

The most widely used supplements of vitamin D are ergocalciferol, cholecalciferol, and calcifediol, with differences in their utilization in European and American countries in part depending on the availability of these compounds, i.e., calcifediol is not available in many countries. Calcifediol is formed by the addition of a 25-hydroxyl group to its precursor, mainly by the enzymes microsomal CYP2R1 and mitochondrial CYP27A1 in the liver [9,15]. Previous studies have shown that calcifediol administration determines a faster increase in circulating 25OHD when compared to cholecalciferol [16,17]. In fact, calcifediol is rapidly absorbed by intestinal cells and transported through the portal vein, hence becoming immediately available in the circulation system. In order to test the efficacy and safety of monthly 0.266 mg calcifediol, Occhiuto et al. performed an open-label investigation with calcifediol administration for two years in post-menopausal women with hypovitaminosis D [18]. The results of this study showed a significant continuous increase in mean 25OHD values even if the mean values at month 24 were not significantly different vs. those at 12 months, but in any case, stable. Similar results were observed for the reduction in mean bone alkaline phosphatase, while no safety concerns were reported. It is worth mentioning that this is the first long-term study with calcifediol administration showing stable and sustained 25OHD concentrations compared to previous investigations that had a much shorter duration, i.e., a few months [19,20].

In addition to the recognized functions in the regulation of calcium absorption, bone remodeling, and bone growth, vitamin D plays a fundamental role as a hormone due to its involvement in numerous enzymatic, physiological, metabolic, and pathophysiological processes in various human organs and systems. There is increasing evidence supporting the role of vitamin D in pancreatic islet dysfunction and insulin resistance in type 2 diabetes, with recent convincing data of the true role of vitamin D supplementation in the prevention of incident type 2 diabetes. However, most meta-analyses evaluating this role have been conducted in people aged 50–60 years [21,22,23,24], with only one focusing on older adults, even if this is the population at highest risk of hypovitaminosis D and type 2 diabetes. Therefore, we conducted an update of the previous systematic review and meta-analysis examining whether hypovitaminosis D could predict incident diabetes in prospective longitudinal studies including older adults [25]. We found that low 25OHD levels were associated with higher incident type 2 diabetes in older adults even after adjusting for relevant potential confounders, confirming and updating the previous meta-analysis conducted in 2017 [26].

Various studies have investigated the association of dietary components and patterns with osteoporosis and bone mineral density in different ethnic groups. However, there are limited studies conducted in Arabian populations. In order to fill this gap, the study by Al-Daghri and collaborators examined whether dietary patterns, and calcium intake in particular, were associated with osteoporosis risk in Saudi Arabian adults [27]. The authors examined cross-sectional data from almost two thousand patients (416 men and 1534 women) with known risk factors for osteoporosis from those attending tertiary hospitals in Riyadh City. The prevalence of osteoporosis was 21.3% and, as expected, was more frequent in women (93.5%) and older participants with lower BMI. The mean calcium intake was only 445.1 mg/day (vs. recommended dietary intake of 1300 mg/day). The consumption of tea was associated with a 20% reduced presence of osteoporosis, while the consumption of fish and eggs was associated with a 10% lower presence of this condition. Conversely, the consumption of biscuits, cakes, and bread slices was significantly associated with a 30% higher presence of osteoporosis. The authors conclude that the finding of extremely low dietary calcium intake among Saudi adults already at risk of osteoporosis suggests an urgent need to promote correction of this alarming dietary deficit in the Saudi adult population.

Aging is a complex and multifactorial process resulting from a number of intrinsic factors and their interactions with the external environment (e.g., air pollutants and radiation, ultraviolet sunlight, physical exercise, diet quality, chemicals in the water), which have become an extensive field of study. In 2013, nine hallmarks of aging were proposed by Lopez-Otin et al., comprising genomic instability, telomere attrition, epigenetic alterations, mitochondrial dysfunction, loss of proteostasis, deregulated nutrient sensing, cellular senescence, stem cell exhaustion, and altered intercellular communication [28], which were expanded in 2023, adding autophagy, microbiome disturbance, and inflammation, among other emerging ones [29,30]. This complex network of cellular and intercellular connections might represent possible targets for the research of agents with pleiotropic effects. Vitamin D has a positive impact not only on skeletal muscle and bone health but also on multiple extra-skeletal organs that express the vitamin D receptor. Thus, vitamin D and its receptor could be molecules potentially targeting the hallmarks of the aging network. In a comprehensive review article, Ruggiero et al. [31] report evidence on the potential effects of vitamin D on the hallmarks of aging, which are still underrepresented in humans and mainly based on pre-clinical models. However, studies in humans seem to confirm the modulatory effect of vitamin D on some of the hallmarks of aging and diseases, opening up new possibilities for future research in this fascinating and useful field.

It is well known that the synthesis of vitamin D_3_ in the human skin is initiated by solar ultraviolet radiation (UVR) exposure of the precursor 7-dehydrocholesterol (7DHC), but the influence of age on this stage of vitamin D metabolism is not completely clear. Borecka et al. performed a prospective standardized study in a group of healthy adults aged ≥ 65 and ≤40 years examining baseline skin 7DHC concentrations and the impact of older age on serum vitamin D_3_ response to solar simulated low-dose UVR to ~35% of the body surface area [32]. The concentration of 7DHC measured in skin biopsies was from unexposed skin (baseline), as well as immediately after UVR exposure and 24 h post-UVR exposure. Blood 25OHD assays were performed at baseline, 24 h, and 7 d post-UVR. Baseline skin 7DHC were similar and not significantly different in younger vs. older adults. Baseline serum 25OHD was also similar in younger vs. older adults and showed significant increases in both groups post-UVR without significant differences between the age groups. Thus, skin 7DHC concentration was not a limiting factor for vitamin D_3_ production in older adults relative to younger adults. These results may help public health guidance on sun exposure and vitamin D nutrition, with particular relevance to the growing populations of healthy older adults. This is in contrast to a previous study of about 40 years ago suggesting that low vitamin D status in older adults was at least partially attributable to lower levels of the skin precursor 7DHC [33]. The authors admit that the main limitation of this study is the relatively low number of volunteers, as intensive in vivo multiple biopsy studies are challenging to recruit a higher number of participants, and this potentially resulted in missed detection of significant differences. Furthermore, the participants were healthy, which prevents the results from being applied to frail older participants with multimorbidity and polypharmacy. Nevertheless, the results are novel and encourage future larger studies to confirm these intriguing results.

Thus, the articles of this Special Issue add important information on the role of two key nutrients, calcium and vitamin D, in human aging and the importance of identifying their deficiency/insufficiency with the aim of implementing an appropriate correction of these deficits.
